# SARS-CoV-2: Advances in Serological Methods and the Understanding of Antibody Escape

**DOI:** 10.3390/ijms24032488

**Published:** 2023-01-27

**Authors:** Daniele Focosi, Fabrizio Maggi

**Affiliations:** 1 North-Western Tuscany Blood Bank, Pisa University Hospital, 56124 Pisa, Italy; 2 National Institute for Infectious Diseases “L. Spallanzani”—IRCCS, 00149 Rome, Italy

In this Special Issue, many original contributions concerning serological methods for SARS-CoV-2 were collected, some of them with implications about therapeutics.

Bonifacio et al. [1] demonstrated that, among four different high-throughput immunoassays (Roche Elecsys® Anti SARS-CoV-2 S, Snibe MAGLUMI® SARS-CoV-2 S-RBD IgG, Snibe MAGLUMI® 2019-nCoV IgG, and EUROIMMUN® SARS-CoV-2 NeutraLISA assays), the NeutraLISA assay displayed the best correlation against the plaque reduction neutralization test (PRNT) gold standard across 83 plasma samples.

Vespa et al. [2] used GSP®/DELFIA® Anti-SARS-CoV-2 IgG immunoassay and flow cytometry TCR-dependent activation-induced marker (AIM) assay (CD134 and CD137) combined with intracellular staining (ICS) for cytokines to show that COVID-19 vaccination or infection generates highly overlapping humoral and cellular immune responses.

Peter et al. from Germany [3] confirmed the risk of immune escape to monoclonal antibody (mAbs) and mAb cocktails. They derived both anti-receptor binding domain (RBD) and anti-N-terminal domain (NTD) mAbs from immunized mice and intriguingly showed how mutations within the RBD can also affect the efficacy of NTD-targeted mAbs.

Da Silva et al. from Luxembourg [4] confirmed the superiority of hybrid immunity over infection-only elicited immunity. In particular, they found that despite lower cumulative anti-Spike antibody levels, the neutralization capability of sera from vaccine breakthrough infections against BA.1 was higher than that of sera from unvaccinated convalescents. These findings are in line with recent data on the potency of COVID-19 convalescent plasma (CCP) from infected and vaccine boosted donors [5,6], likely acknowledging the benefits of heterologous immunity and epitope spreading.

Cia et al. from Belgium [7] performed an important in silico study to show that the class of nAbs that contributes most to the post-infection immunity (but not to the post-mRNA-1273 immune response) can bind the spike protein in its closed conformation; while this only partially inhibits ACE2 binding, this class blocks transition to an open conformation.

Wang et al. from Taiwan [8] provided an important upgrade for pseudoviral neutralization assays. They showed that the presence of envelope (E) and membrane (M) proteins in addition to the Spike protein in pseudovirions increased infectivity by promoting the S protein priming. This improved mimicry of the actual PRNT could make pseudoviral neutralization assays more reliable.

Finally, we and colleagues from Johns Hopkins University systematically reviewed current knowledge on immune escape capabilities of SARS-CoV-2 against both anti-Spike mAbs and COVID-19 convalescent plasma (CCP) [9], a resource that is increasingly important for the treatment of immunocompromised patients. As expected, the polyclonal product was more escape-resistant than mAbs.

## References

[1] Bonifacio M.A., Laterza R., Vinella A., Schirinzi A., Defilippis M., Di Serio F., Ostuni A., Fasanella A., Mariggiò M.A. (2022). Correlation between In Vitro Neutralization Assay and Serological Tests for Protective Antibodies Detection. Int. J. Mol. Sci..

[2] Vespa S., Simeone P., Catitti G., Buca D., De Bellis D., Pierdomenico L., Pieragostino D., Cicalini I., Del Boccio P., Natale L. (2022). SARS-CoV-2 and Immunity: Natural Infection Compared with Vaccination. Int. J. Mol. Sci..

[3] Peter A.S., Grüner E., Socher E., Fraedrich K., Richel E., Mueller-Schmucker S., Cordsmeier A., Ensser A., Sticht H., Überla K. (2022). Characterization of SARS-CoV-2 Escape Mutants to a Pair of Neutralizing Antibodies Targeting the RBD and the NTD. Int. J. Mol. Sci..

[4] da Silva E.S., Kohnen M., Gilson G., Staub T., Arendt V., Hilger C., Servais J.-Y., Charpentier E., Domingues O., Snoeck C.J. (2022). Pre-Omicron Vaccine Breakthrough Infection Induces Superior Cross-Neutralization against SARS-CoV-2 Omicron BA.1 Compared to Infection Alone. Int. J. Mol. Sci..

[5] Sullivan D.J., Franchini M., Joyner M.J., Casadevall A., Focosi D. (2022). Analysis of anti-Omicron neutralizing antibody titers in different convalescent plasma sources. Nat. Comm..

[6] Sullivan D.J., Franchini M., Senefeld J.W., Joyner M.J., Casadevall A., Focosi D. (2022). Plasma after both SARS-CoV-2 boosted vaccination and COVID-19 potently neutralizes BQ1.1 and XBB. bioRxiv.

[7] Cia G., Pucci F., Rooman M. (2022). Analysis of the Neutralizing Activity of Antibodies Targeting Open or Closed SARS-CoV-2 Spike Protein Conformations. Int. J. Mol. Sci..

[8] Wang H.-I., Chuang Z.-S., Kao Y.-T., Lin Y.-L., Liang J.-J., Liao C.-C., Liao C.-L., Lai M.M.C., Yu C.-Y. (2021). Small Structural Proteins E and M Render the SARS-CoV-2 Pseudovirus More Infectious and Reveal the Phenotype of Natural Viral Variants. Int. J. Mol. Sci..

[9] Focosi D., Maggi F., Franchini M., McConnell S., Casadevall A. (2022). Analysis of Immune Escape Variants from Antibody-Based Therapeutics against COVID-19: A Systematic Review. Int. J. Mol. Sci..

